# Concurrent Intraventricular and Sacral Spinal Drop Metastasis of Ganglioglioma in an Adult Patient: A Case Report and Review of Literature

**DOI:** 10.7759/cureus.538

**Published:** 2016-03-22

**Authors:** Hasan R Syed, Jay W Rhee, Ribhu T Jha, Daniel Felbaum, Christopher G Kalhorn

**Affiliations:** 1 Neurosurgery, Medstar Georgetown University Hospital; 2 Neurosurgery, Holy Cross Hospital

**Keywords:** ganglioglioma, sacral, intraventricular, drop metastasis, spinal metastasis

## Abstract

Gangliogliomas are uncommon tumors of the central nervous system and rarely occur in the lateral ventricle or present with drop metastasis. We report a 49-year-old male who presented with a six-week history of left leg pain and numbness. Clinical examination revealed no focal neurological deficits. Magnetic resonance imaging (MRI) demonstrated enhancing nodular lesions in the sacral spine abutting the S2 nerve root. Further imaging of the neuroaxis demonstrated a cystic lesion in the left frontal horn of the lateral ventricle. Gross total surgical resection of the ventricular lesion was performed through a transcortical approach, followed by resection of the sacral spinal drop metastasis in a staged manner. A histopathological analysis revealed the diagnosis of low-grade ganglioglioma. To our knowledge, this is the first reported case of a low-grade intraventricular ganglioglioma presenting with symptoms associated with drop metastasis in an adult patient.

## Introduction

Gangliogliomas are slow-growing, generally benign tumors of the central nervous system (CNS) that consist of both glial and ganglion cells. The incidence is 0.4% to 0.7% of all CNS tumors [1], 0.4% to 7.6% of primary brain tumors [2-3], and only 1% of all spinal cord tumors [4], of which most are located within the cervical and thoracic spinal cord [5]. These tumors tend to occur in children and young adults under the age of 30 [6]. The most common location for these tumors is the temporal lobe of the brain, but they have been reported to occur in locations throughout the CNS including frontal, parietal, and occipital lobes, and rarely in the brain stem, optic chiasm, pineal region, and spinal cord [7-8]. This tumor is rarely found in the lateral ventricle. To our knowledge, there are only seven prior case reports of gangliogliomas in this location [6, 9-14]. Furthermore, few cases of leptomeningeal spread of low grade gangliogliomas have been described in pediatric patients [15-17]. Here we report a rare case of concomitant intraventricular and sacral intradural gangliogliomas in an adult male. Informed consent was obtained from the patient for this study.

## Case presentation

### History of present illness

A 49-year-old male presented in January 2013 with a six-week history of excruciating left leg pain that radiated down the back of his thigh and anterior portion of the leg. The pain was sharp, constant, and refractory to pain medications. The patient also endorsed numbness over the left shin but denied any focal weakness in his lower extremities, or bowel or bladder dysfunction. He required a wheelchair secondary to severe pain for 48 hours prior to presentation in the emergency department. His past medical history was significant for four vessel coronary artery bypass graft surgery in 2000, diabetes, and carpel tunnel release surgery. On physical exam, the patient was neurologically intact with full strength in bilateral upper and lower extremities.

### Imaging studies

**Figure 1:**
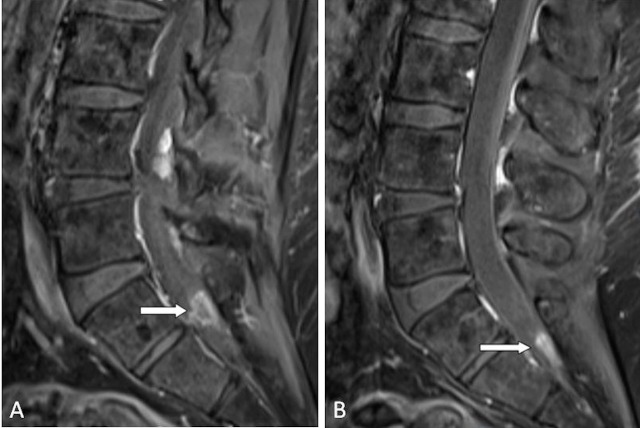
Preoperative MRI Spine Parasagittal gadolinium enhanced T1-weighted MRI demonstrating an intradural lesion at the level of S1-2 abutting the left S2 nerve root (A) and at the level of S2 (B).

**Figure 2:**
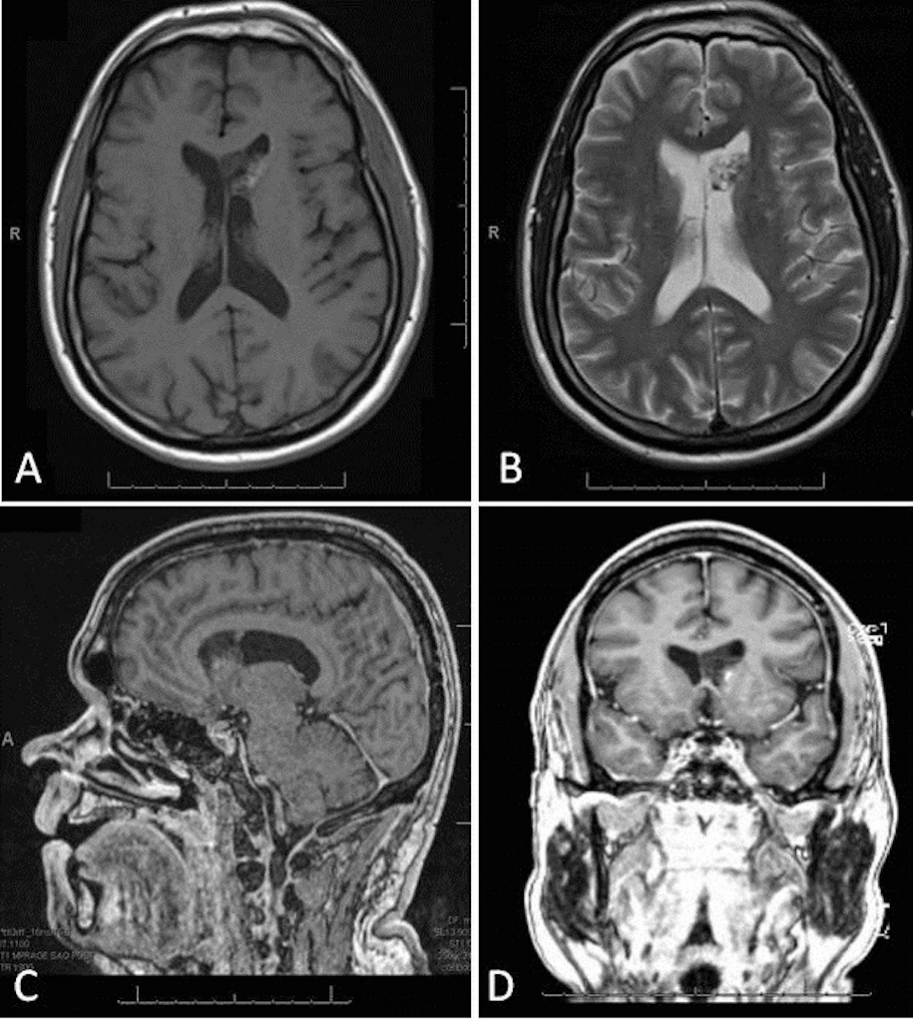
Preoperative MRI Brain Axial T1- (A) and T2- (B) weighted MRI demonstrating a cystic-like lesion within the left frontal horn of the lateral ventricle. Part of the lesion is hyperintense to grey matter on the T1-weighted images and hypointense on the T2 images. Sagittal (C) and coronal (D) gadolinium enhanced T1-weighted MRI showing no definite tumor enhancement.

A contrasted MRI of the lumbar spine revealed enhancing nodular lesions located intradurally at S1-S2 and S2 (Figure 1). The S1-S2 nodule measured 1.3 cm x 1.1 cm x 0.7 cm and was abutting the descending left S2 nerve root. The S2 nodule measured 1.2 cm x 0.7 cm x 0.3 cm. The characteristics and locations of the lesions were concerning for drop metastasis. Therefore, MRI scans of the brain and cervical and thoracic spine were obtained. This revealed a 2 cm x 1.8 cm lesion in the left frontal horn of the lateral ventricle (Figure 2). The lesion appeared mostly cystic and was partly hyperintense on T1-weighted images, likely representing calcification, and hypointense on T2-weighted MRI. There was no definite enhancement noted on T1-weighted post-contrast images. A computed tomography (CT) scan of the chest, abdomen, and pelvis was negative for a primary source of malignancy. The pre-surgical radiologic differential diagnosis was most likely ependymoma or neurocytoma.

### Treatment and clinical course

The patient underwent a left frontal craniotomy for a transcortical, transventricular approach for resection of the left frontal horn intraventricular tumor. A bicoronal incision was made extending anterior to the left tragus across the midline to above the right ear. After superficial dissection, a left-sided craniotomy was completed, and the dura was opened in a curvilinear manner and reflected towards the midline. Trajectory to the left frontal horn of the lateral ventricle was verified by neuro-navigation. The left frontal horn was approached by performing a corticectomy. The tumor was readily visualized upon entering the ventricle. The mass was a grossly grayish appearing tumor attached to the left caudate head. Several pieces of tumor were harvested and sent for intraoperative consultation. Review of the specimens by neuropathologists showed the tumor to be glial in origin with proliferation of ganglion cells. Gross total resection of the tumor was achieved utilizing bipolar electrocautery and gentle suction. An external ventricular drain (EVD) was placed through the corticectomy tract into the left frontal horn. Hemostasis was achieved, and the craniotomy was closed in standard fashion.

Postoperatively the patient was taken to the neurosurgical intensive care unit. The EVD was gradually weaned, and the patient was transferred to a floor bed. He was eventually discharged home with scheduled follow-up for resection of the sacral intradural tumors.

The patient returned to the hospital two weeks following discharge for resection of S1 and S2 intradural tumors. An incision was performed in the midline at the level of the sacrum. Intraoperative fluoroscopy was used to localize the appropriate level. Laminectomies were performed at S1 and S2 to expose the thecal sac. Intraoperative ultrasound was then employed to visualize the tumors. The dura was opened and two densely calcified nodules were visualized; one on the left at the level of S1, and a smaller nodule on the right at the level of S2. Fragments of the nodules were sent for an intraoperative consultation, and the results revealed characteristics of a spindle cell tumor with prominent calcification and ganglion cells. Gross total resection of the left S1 nodule was achieved. However, because the right S2 nodule was completely blended in with a number of rootlets, this tumor was debulked but not completely resected. The surgical site was closed in standard fashion. The patient was admitted to a floor bed and was monitored for cerebrospinal fluid (CSF) leak for 48 hours. He was then allowed to ambulate. He remained neurologically intact with full strength and was discharged home with scheduled follow-up.

### Pathology

**Figure 3:**
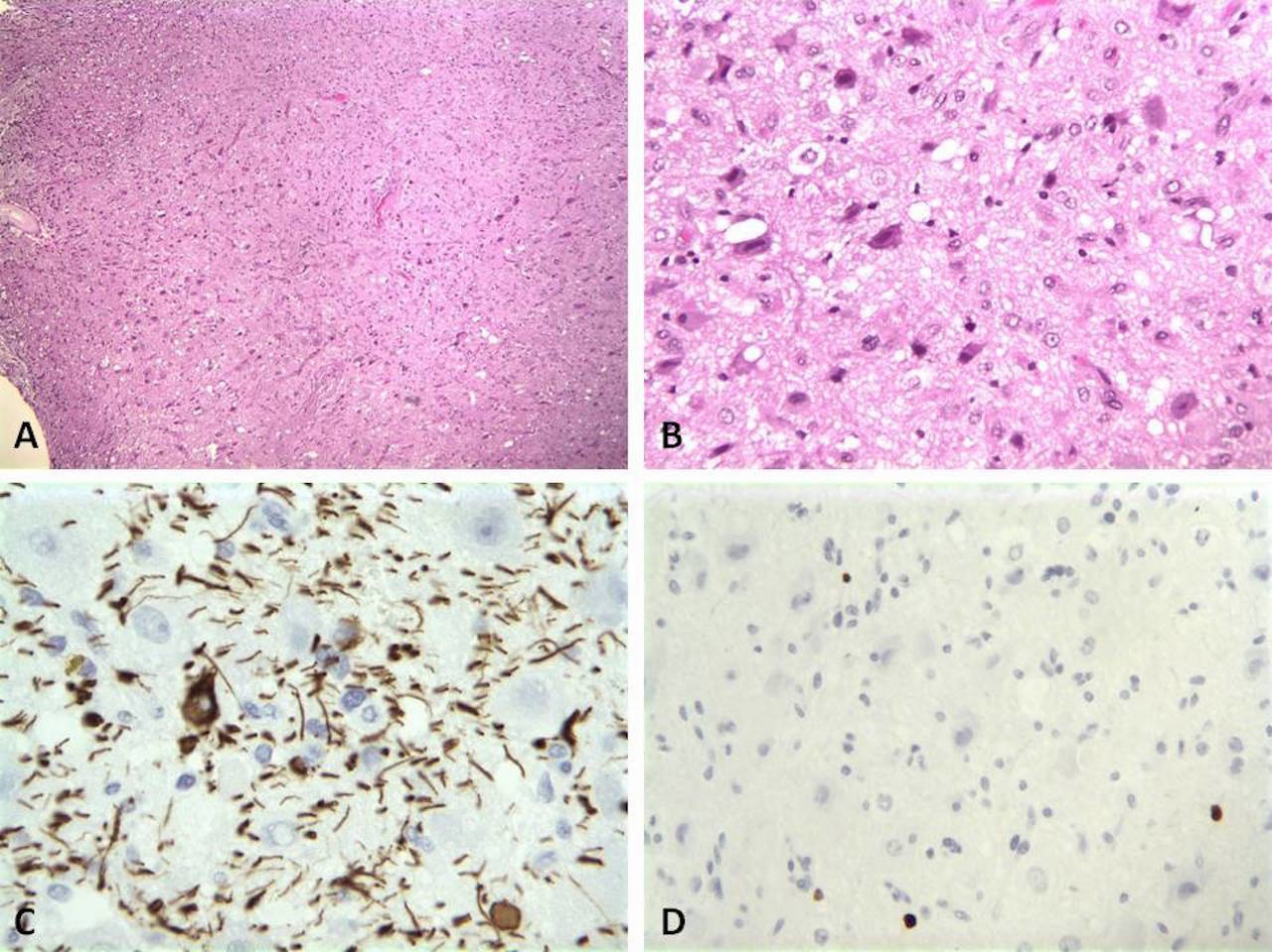
Intraventricular Pathology Slides Intraventicular ganglioglioma seen at low power magnification (A, H&E stain, 40X); High power magnification with abundant ganglion cell proliferation (B, H&E stain, 200X); GFAP positive glial cells (C, GFAP stain, 400X), and low MiB-1 index (D, Ki-67 stain, 400X).

**Figure 4:**
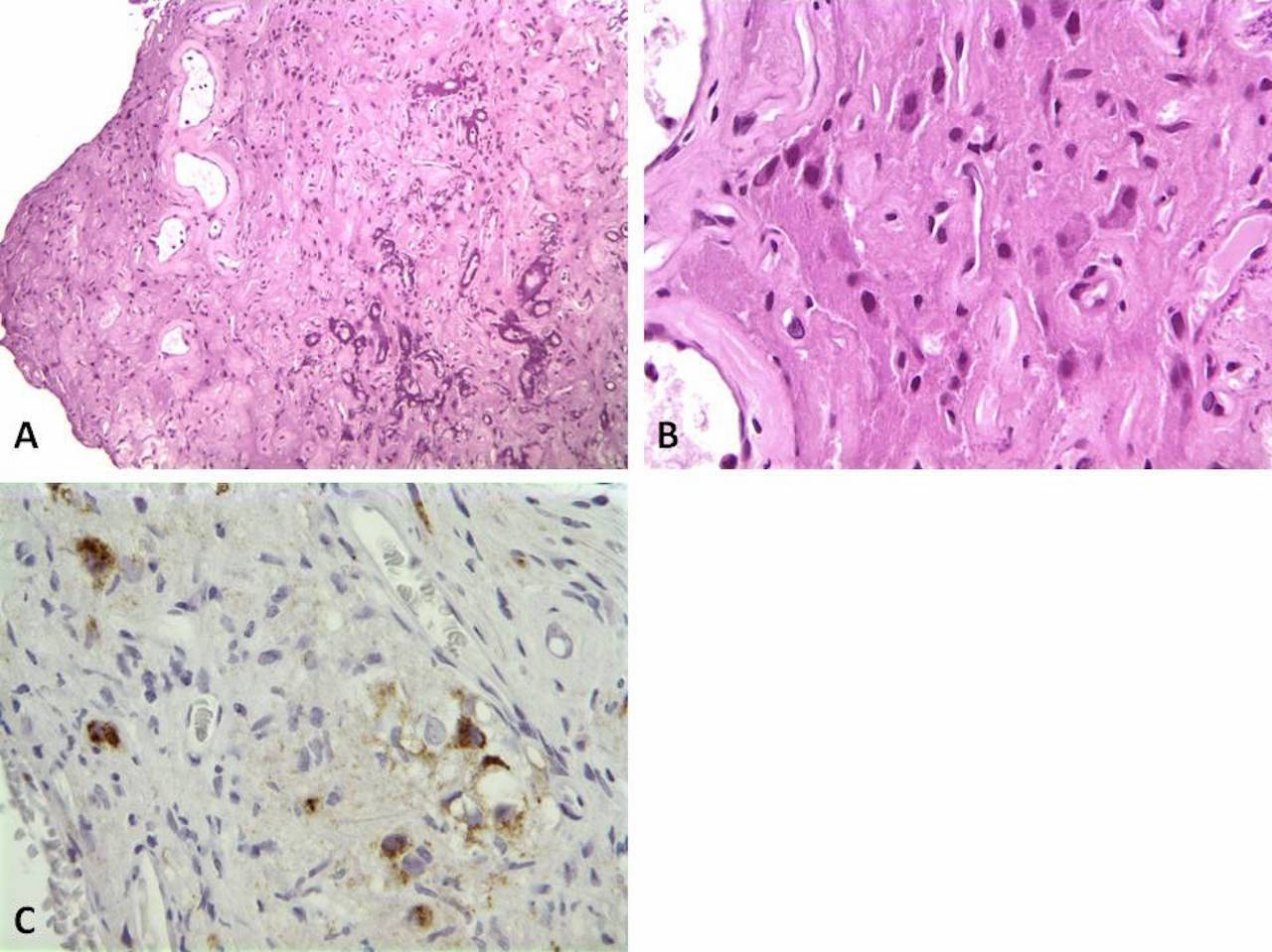
Sacral Spinal Pathology Slides Sacral drop metastasis with abundant calcifications (A, H&E stain, 40X); High power view of the sacral tumor (B, H&E stain, 400X), and Chromogranin stain highlighting the ganglion cells within the sacral tumor (C, Chromogranin stain, 400X).

The permanent sections of the intraventricular mass revealed a glial tumor containing numerous ganglion and glial cells, some with clear cytoplasm but without significant atypia. No necrosis, or vascular proliferation was apparent. Immunohistochemical analyses were performed which showed the glial cells to be positive for chromogranin, neurofilament, and weakly positive for calretinin. Other markers like glial fibrillary acidic protein (GFAP) and S100 highlighted few spindled cells. The MIB-1 proliferative index using Ki-67 immunohistochemical stain was low at a value of 3% (Figure 3). These results supported the diagnosis of WHO grade II ganglioglioma. Similarly the permanent sections of the sacral nodules revealed GFAP-positive spindle cells intermixed with chromogranin positive ganglion cells (Figure 4). Interestingly, the MIB-1 proliferative index was low as well. These results support the diagnosis of low-grade ganglioglioma consistent with drop metastasis from the known intraventricular ganglioglioma.

## Discussion

This report describes the rare occurrence of a ganglioglioma of the left lateral ventricle and is the first report of such an intracranial mass exhibiting features of metastasis to the sacral spine in a 49-year-old male. Review of literature found only seven reported cases of lateral ventricle ganglioglioma [12] and one reported case of a sacral spine ganglioglioma [18]. Five of the patients with lateral ventricle gangliogliomas were female [6,9,11-12,14] while two were males [10,13]. Five of the seven patients reported presenting symptom of headache [9-12,14] while two presented with seizures [6,13]. Gross total resection was achieved in four of the seven patients [9-10,12,14], subtotal resection was achieved in two of the seven patients [6,13], and the degree of resection was not clearly stated in one case report [11]. None of these cases of intraventricular gangliogliomas report any radiologic images of the spine, likely because the patients did not have any concerning symptoms for spine pathology.

While the review of literature reveals several reports describing intramedullary spinal gangliogliomas of the cervical, thoracic, and conus medularis regions [1,5,19], only one is found reporting this lesion in the sacral spine [18]. Nonetheless, there are no reports of gangliogliomas of the brain disseminating to the spine.

Gangliogliomas are rare low-grade neoplasms defined by the presence of dysplastic neuronal and glial cells that were first described by O.C. Perkins in 1926 [20]. They are tumors that are most often classified as WHO grade I or II and present with a 10-year survival rate of 82-93% [19]. Overall, gangliogliomas encompass only 0.33% to 1.3% of adult intracranial neoplasms [19,21,22] and only 1% of adult spinal neoplasms [4]. Most spinal gangliogliomas are located within the cervical and thoracic cord [5].

The most common presenting symptom of intracranial ganglioglioma is a seizure, with an incidence rate of 65-92% [1,7,19]. Gangliogliomas of the ventricular system are more likely to present with headaches, with an incidence of 67%. A retrospective study of 56 patients with spinal cord ganglioglioma documented that 50% of patients presented with paraparesis and 46% presented with segmental pain [5]. A previously reported case of a rare sacral spine ganglioglioma documented presenting symptoms of progressive bowel and bladder dysfunction, lower extremity weakness, and sensory abnormalities in the sacral dermatomes [18]. Radiographically, gangliogliomas can present with significant heterogeneity. A review of 48 gangliogliomas on CT described 78% of these masses as having at least some areas of low density [23]. The majority (52%) of gangliogliomas have cystic and solid radiographic features; 43% are radiographically entirely solid and 5% are entirely cystic [24,25]. T1-weighted MRI findings of these tumors are not pathognomonic as there is much variability with regards to intensity [26]. The majority (68-92%) of gangliogliomas are hyperintense on T2-weighted MRI scans [24,26].

## Conclusions

We report the first case of intraventricular ganglioglioma with sacral spinal metastasis in an adult patient. While the patient’s symptoms were only located in the lower extremity, radiological images were suspicious for drop metastasis. In this case, cranial imaging demonstrated an intraventricular lesion. The authors emphasize the importance of a complete evaluation in such cases where suspicion for drop metastasis exists.

## References

[1] Lang FF, Epstein FJ, Ransohoff J (1993). Central nervous system gangliogliomas. Part 2: Clinical outcome. J Neurosurg.

[2] Hirose T, Scheithauer BW, Lopes MB (1997). Ganglioglioma: an ultrastructural and immunohistochemical study. Cancer.

[3] Majós C, Coll S, Aguilera C (2000). Intraventricular mass lesions of the brain. Eur Radiol.

[4] Nass R, Whelan MA (1981). Gangliogliomas. Neuroradiology.

[5] Jallo GI, Freed D, Epstein FJ (2004). Spinal cord gangliogliomas: a review of 56 patients. J Neurooncol.

[6] Matsumoto K, Tamiya T, Ono Y (1999). Cerebral gangliogliomas: clinical characteristics, CT and MRI. Acta Neurochir (Wien).

[7] Haddad SF, Moore SA, Menezes AH (1992). Ganglioglioma: 13 years of experience. Neurosurgery.

[8] Louis DN, Ohgaki H, Wiestler OD (2007). The 2007 WHO classification of tumours of the central nervous system. Acta Neuropathol.

[9] Jaeger M, Hussein S, Schuhmann MU (2001). Intraventricular trigonal ganglioglioma arising from the choroid plexus. Acta Neurochir.

[10] Majós C, Aguilera C, Ferrer I (1998). Intraventricular ganglioglioma: case report. Neuroradiology.

[11] Nair V, Suri VS, Tatke M, Saran RK, Malhotra V, Singh D (2004). Gangliogliomas: a report of five cases. Indian J Cancer.

[12] Russell D, Rubinstein L (1962). Ganglioglioma: a case with long history and malignant evolution. J Neuropathol Exp Neurol.

[13] Silver JM, Rawlings CE, Rossitch E (1991). Ganglioglioma: a clinical study with long-term follow-up. Surg Neurol.

[14] Yin Foo Lee G, Scott G, Blumbergs PC (2001). Ganglioglioma of the lateral ventricle presenting with blepharospasm - case report and review of the literature. J Clin Neurosci.

[15] Hukin J, Siffert J, Velasquez L (2002). Leptomeningeal dissemination in children with progressive low-grade neuroepithelial tumors. Neuro-oncol.

[16] Massimi L, Battaglia D, Paternoster G (2009). Segmental spinal myoclonus and metastatic cervical ganglioglioma: an unusual association. J Child Neurol.

[17] Bell WO, Packer RJ, Seigel KR (1988). Leptomeningeal spread of intramedullary spinal cord tumors. Report of three cases. J Neurosurg.

[18] Smith SJ, Lownie SP, Duggal N (1998). Case of the month: April 1998 - 30 year old male with perineal numbness. Brain Pathol.

[19] Hakim R, Loeffler JS, Anthony DC (1997). Gangliogliomas in adults. Cancer.

[20] Johnson JH Jr, Hariharan S, Berman J, Sutton LN, Rorke LB, Molloy P, Phillips PC (1997). Clinical outcome of pediatric gangliogliomas: ninety-nine cases over 20 years. Pediatr Neurosurg.

[21] Blümcke I, Wiestler OD (2002). Gangliogliomas: an intriguing tumor entity associated with focal epilepsies. J Neuropathol Exp Neurol.

[22] Demierre B, Stichnoth FA, Hori A (1986). Intracerebral ganglioglioma. J Neurosurg.

[23] Dorne HL, O’Gorman AM, Melanson D (1986). Computed tomography of intracranial gangliogliomas. Am J Neuroradiol.

[24] Zentner J, Wolf HK, Ostertun B (1994). Gangliogliomas: clinical, radiological, and histopathological findings in 51 patients. J Neurol Neurosurg Psychiatry.

[25] Zhang D, Henning TD, Zou LG (2008). Intracranial ganglioglioma: clinicopathological and MRI findings in 16 patients. Clin Radiol.

[26] Castillo M, Davis PC, Takei Y (1990). Intracranial ganglioglioma: MR, CT, and clinical findings in 18 patients. AJNR Am J Neuroradiol.

